# Identifying a stable bulk dmLT adjuvant formulation at a clinically relevant concentration

**DOI:** 10.1016/j.vaccine.2023.01.005

**Published:** 2023-02-10

**Authors:** Marcus R. Estrada, Anan Bzami, Elizabeth B. Norton, Jessica A. White

**Affiliations:** a PATH, 2201 Westlake Ave, Seattle, WA 98122, United States; b Department of Microbiology and Immunology, Tulane University School of Medicine, 1430 Tulane Avenue, New Orleans, LA 70112, United States

**Keywords:** dmLT, Adjuvant, Formulation, *E.coli* heat-labile toxin

## Abstract

Double mutant heat-labile toxin (dmLT) is a novel vaccine adjuvant under development with several different vaccine candidates. Studies using existing dmLT adjuvant stocks require significant dilution to achieve a clinically relevant dose. This dilution leads to wastage of the adjuvant. This manuscript describes a limited formulation study to improve the stability of bulk dmLT at a more clinically relevant concentration (20 µg/mL) with minimal changes to the existing bulk dmLT formulation. *In vitro* methods were used to evaluate dmLT stability after lyophilization and short-term accelerated stability studies. The addition of the excipient polysorbate 80 (PS80) at 0.05 % to the existing dmLT formulation was identified as the lead modification that provided improved stability of the lyophilized dmLT at 20 µg/mL through 4 weeks at 40 °C.

## 1 Introduction

Double mutant heat-labile toxin (dmLT) is derived from *Escherichia coli* (*E. coli*) heat-labile toxin (LT) and consists of an enzymatic A-subunit and a pentameric B-subunit with two amino acid substitutions in the A-subunit [1], [2], [3]. dmLT has demonstrated safety in several human clinical studies [4], [5], [6], [7]. A low dose of dmLT, for example, a 0.50-µg dose, has been used in Phase 1 human clinical studies [7], [8].

dmLT is currently lyophilized at 1 mg/mL, requiring reconstitution and dilution prior to use (∼1 µg/mL typical formulation concentration). This dilution leads to a significant amount of dmLT wastage. Additionally, dmLT may be unstable or lost to vessel walls during the dilution process [10]. To support future use, improve stability, and reduce wastage of dmLT, this study was conducted to identify a lyophilized formulation that would maintain a more clinically relevant and useable bulk dmLT concentration (target of 20 µg/mL) and significantly reduce waste [9]. dmLT stability at elevated temperature was evaluated using *in vitro* characterization methods, enzyme-linked immunosorbent assay (ELISA), sodium dodecyl sulfate–polyacrylamide gel electrophoresis (SDS-PAGE) gel analysis, western blot (WB), and size-exclusion high-performance liquid chromatography (SEC) [10]. Animal studies were conducted using inactivated polio vaccine and two enterotoxigenic *E. coli* (ETEC) vaccine candidates as model vaccine antigens to evaluate if altering the dmLT formulation had any effect on adjuvanticity or immunologic outcomes. Results from the animal studies are being submitted for publication in a separate manuscript by Dr. Norton’s laboratory from Tulane University.

Minor changes to the dmLT formulation (buffer, sugar, and detergent) at the selected target stock concentration of 20 µg/mL were evaluated. Formulations were selected based on both previously published studies and on the existing dmLT formulation [11]. This work deliberately sought to limit changes to the existing dmLT formulation because of the extensive preclinical and clinical data in the existing formulation. The addition of the excipient polysorbate 80 (PS80) at 0.05 % (w/v) was identified as the most significant improvement to dmLT stability at 20 µg/mL and provided minimal changes to the existing formulation.

## 2 Materials and methods

Lyophilized dmLT in 3-mL glass vials stored at –30 °C produced by the Walter Reed Army Institute of Research Pilot Bioproduction Facility (Silver Spring, MD, lot #1735, technical batch manufactured on November 21, 2011) were used for this work. Vials contained 700 μg of protein in 42.7 mM sodium phosphate, 10.7 mM potassium phosphate, 82 mM NaCl, 5 % lactose monohydrate, pH 7.4.

Formulation evaluations focused on three separate components: stabilizing buffer (phosphate, citrate, and histidine), bulking sugars (sucrose, lactose, and trehalose), and additional stabilizing excipients (methionine and PS80) [11]. dmLT formulations were prepared by diluting dmLT reconstituted to 1 mg/mL with the selected test formulation to achieve a final dmLT concentration of 20 µg/mL. The 20 µg/mL concentration was selected based on ongoing preclinical and clinical studies that targeted a final concentration of 1 µg/mL. A bulk concentration of 20 µg/mL allows for addition of dmLT to a vaccine formulation in one step. This concentration would also cover a range of possible clinical study doses and be concentrated enough to allow robust analytical testing. This dilution resulted in a 50-fold reduction of the residual bulk dmLT formulation components present in the test formulations. Formulations were also prepared to 150 ug/mL dmLT by diluting dmLT bulk 6.7-fold with the selected formulation buffer containing 0.05 % PS80.

dmLT formulations were prepared as described below using the following excipients: citric acid monohydrate (VWR cat# RB908), histidine (J.T.Baker cat# 2080-05), sucrose (J.T.Baker cat# 4074-01), sodium phosphate dibasic (Macron cat# 7774-04), sodium chloride (NaCl; J.T.Baker cat# 3628-01), potassium phosphate monobasic (Macron cat# 7746-04), phosphoric acid (J.T.Baker cat# 0268-00), trehalose dehydrate (Pfanstiehl, Inc. cat #T-104-4), lactose monohydrate (Spectrum Chemical cat # LA105), polysorbate 80 (PS80, J.T.Baker Cat# 4117-02), methionine (Sigma-Aldrich cat# M5308), sodium hydroxide (EMD Millipore cat# 1.09137.1000), and hydrochloric acid (Sigma-Aldrich cat# 320331).

dmLT stability was evaluated with citrate pH 6.0, histidine pH 6.5, at phosphate pH 7.0 buffers with 150 mM NaCl and 0.02 % PS80 at 20 µg/mL dmLT. Citrate buffer was prepared at 20 mM, histidine buffer was prepared at 20 mM and phosphate buffer was prepared at 20 mM and pH adjusted with HCl or NaOH. dmLT samples in each buffer were held at 25 °C and 37 °C for up to 7 days.

Formulations were lyophilized in 2-mL glass vials (West Pharmaceutical Services, cat# 68000314) and filled with 0.5 mL formulation, and 13-mm lyophilization FluoroTec stoppers (West Pharmaceutical Services, cat# 19700034) were placed on vials. Vials were transferred to a Millrock LD85 freeze dryer, set to 4 °C for 2 h, and ramped to –45 °C in 2 h and held for 8 h. The pressure was lowered to 100 mTorr, and the shelf was ramped to –20 °C in 1 h and held for 23 h. The pressure was increased to 200 mTorr, and the shelf was ramped to 5 °C in 1 h and held for 5 h, and then ramped to 30 °C in 0.5 h and held for 5 h. The temperature was dropped to 4 °C, then the chamber was filled with nitrogen gas and the vials were stoppered.

Accelerated stability samples of lyophilized dmLT formulations containing 53.4 mM phosphate, 0.05 % PS80, and 5 %, 7.5 %, or 10 % lactose were held at 40 °C for 1 month.

The effect of heat and low pH were evaluated in liquid formulations containing dmLT at 20 µg/mL and 150 µg/mL. Samples were subjected to 40 °C at neutral pH or low pH buffer at 2–8 °C for 3 days. Low pH buffer was prepared by diluting 1 mg/mL dmLT to 100 ug/mL with pH 2.4 phosphate-buffered saline (PBS) containing 0.05 % PS80 to achieve a pH of 4.0 +/– 0.2. The buffer was neutralized after 3 days with pH 10.8 PBS supplemented with 0.05 % Tween 20 (PBST) to achieve a final pH of 7.0 +/0.2 prior to testing.

A monoclonal antibody (mAb; Precision Antibody, Columbia, MD) was developed against the A-subunit of dmLT for ELISA use. Mice were immunized with purified A-subunit and dmLT antigen. Sera was screened against dmLT, dmLT bound to monosialotetrahexosylganglioside (GM1; cellular receptor for dmLT), and A- and B-subunits [10]. The LT neutralizing capacity of selected mAbs were evaluated at University of Tulane as described previously [12]. mAb (2D8) demonstrated LT neutralizing activity and was selected for use in the dmLT ELISA.

A sandwich ELISA for dmLT was conducted based on previously published methods with the following modifications [10], [13]. Briefly, the mAb, 2D8, was used for detection of intact dmLT molecules bound to GM1. Detection antibody (Goat Anti-Mouse IgG γ chain cat. AP503P) was prepared by diluting 1:8,000 in Dulbecco’s phosphate-buffered saline (DPBS; Fisher Scientific cat. SH30378.02) with 1 % bovine serum albumin (w/v) (Thermo Fisher Scientific cat. J10857) and 0.05 % Tween 20 (w/v) (Fisher Scientific cat. 337-500).

SEC was used to evaluate dmLT stability because it allows for measurement of intact dmLT molecules (AB5) as well as any combinations of subunits in the mixture. SEC was performed using the following method: a mobile phase of 50 mM sodium phosphate and 500 mM arginine HCl (Sigma-Aldrich cat # A4599) pH 6.8 was used along with a ProSEC 300S 300 × 7.5 mm column (Agilent cat. PL1147-6501) and a ProSEC 300S Guard column 50 × 7.5 mm (Agilent cat. PL1147-1501). A flow rate of 0.5 mL/min over 45 mins was used with ultraviolet (UV) detection at 280 nm and fluorescence detection at excitation 280 nm/emission 335 nm. Samples were injected at 100 µl volumes in duplicate. Multiple injections of a 500 µg/mL dmLT standard with an injection volume of 10 µl were included in every experiment. Peaks were collected from 150 µg/mL dmLT sample injections into tubes containing 0.1 % PS80 in saline, starting at 18.8 min at 0.4-minute increments and tested by ELISA, WB, and SDS-PAGE. Experiments were conducted using Dionex UltiMate 3000 high-performance liquid chromatography (HPLC) with Chromeleon console software version 7.2. Results are shown as the percent of the main peak measured by SEC.

SDS-PAGE and WB analysis were conducted using Novex Blot SDS gels (Invitrogen Thermo Fisher Scientific cat. NP0322BOX). Samples were prepared using NuPAGE 4X LDS (lithium dodecyl sulfate) sample buffer (Thermo Fisher Scientific cat. NP0007) and heated at 90 °C for 5 min. 20 µl of each sample was loaded per well and run at 100 V for 90 min (Life Technologies PowerEase 90 W system) with Bolt running buffer (Life Technologies cat. B0002). SDS-PAGE gels were stained by rinsing the gel in deionized (DI) water and then incubating for 60 min using PageBlue protein staining solution (Thermo Fisher Scientific cat. 24620). Gels were de-stained in DI water overnight. WB transfer was done at 30 V for 60 min in transfer buffer (Novex Life Technologies cat. BT00061) and 10 % methanol (Sigma-Aldrich cat. R178748). Polyvinylidene difluoride (PVDF) membranes (Life Technologies cat. LC2002) were soaked in methanol before being soaked in transfer buffer and assembling the WB sandwich. After transfer was completed, blots were blocked with casein buffer (Thermo Fisher cat. 37528) for 30 min at room temperature (RT). Blots were then incubated for 60 min at RT with anti-rabbit A-subunit sera, diluted in casein buffer. After, incubation blots were washed with PBS containing 0.05 % polysorbate 20 (Teknova cat. P31898). Bound primary antibody was detected using goat anti-rabbit secondary antibody (Invitrogen cat. A32733) diluted in casein buffer and incubated for 60 min at RT. Blots were washed before images were captured (Syngene G: Box mini gel system wet scan at 647 nm).

Animal studies were performed at Tulane University and approved by the Tulane University Institutional Animal Care and Use Committee. Inactivated polio vaccine, IPOL 80DU/ml (Sanofi Pasteur) was purchased from Tulane Pharmacy and combined with dmLT diluted in Media-199 (Gibco). Female BALB/c mice, 6–8 weeks of age, were purchased from Jackson Laboratories (Strain #:000651) and housed in sterile cages. Formulations were injected with a 0.3 cc needle into the shaved, hind flank skin directly into the caudal thigh muscle (IM), alternating right or left sides with each immunization. Immunizations were performed 3 times at a 3-week interval. Mice were euthanized with CO2 and exsanguinated for blood collection at week 5. Blood was centrifuged for serum and subsequently stored at −20 °C until analysis. Samples were tested using a standard microneutralization assay for antibodies to oral poliovirus types 1, 2, and 3 Sabin strains (kindly provided from Dr. Steven Oberste, Polio and Picornavirus Laboratory Branch Chief, CDC). Protocols were adopted from established protocols at the Global Polio Specialized Laboratory, Centers for Disease Control and Prevention [40]. Briefly, 80–100 CCID50 of each poliovirus serotype and 2-fold serial dilutions of serum were combined and pre-incubated at 35 °C, 5 % CO2 for 3 h before addition of HEp-2(C) cells (50,000 cells per well) in 96-well tissue culture treated flat bottom plates (CytoOne). After incubation for 5 days at 35 °C and 5 % CO2, plates were stained with Neutral Red (Sigma) and imaged by Immunospot ELISPOT Analyzer. Each specimen was run in duplicates, with parallel specimens from one study subject tested in the same assay run, and the neutralization titers estimated by the Spearman-Kärber method and reported as the reciprocal of the calculated 50 % end point titer (e.g. serum dilution factor) and log2 transformed. Reference antiserum pool was used to monitor assay performance and variation. A serum sample was considered positive if antibodies were present at ≥ 1:8 dilution. Specimens with antibody titers < 1:8 were considered seronegative and given a log2 value of 2.

## 3 Results

**Fig. 1:**
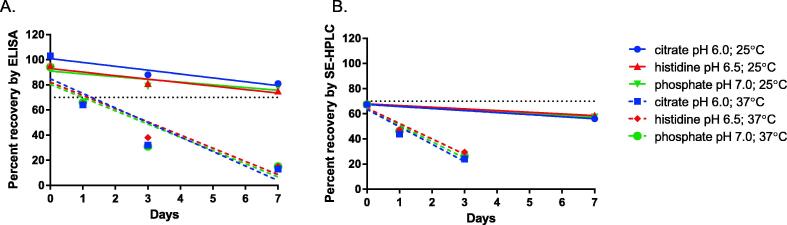
Buffer compatibility with dmLT. dmLT stability was evaluated with citrate, histidine, at phosphate buffers at a dmLT concentration of 20 µg/mL. Samples of each buffer were held at 25 °C and 37 °C for up to 7 days and tested by ELISA and SEC at t = 0-, 1-, 3-, and 7-day time points.

dmLT stability in alternate buffers, citrate and histidine, were compared to the existing phosphate buffer. As seen in Fig. 1, there was no difference in dmLT stability at 20 µg/mL in any of the buffers tested. The most significant effect on dmLT stability was temperature regardless of the buffer being tested. These findings do not support changing from the existing phosphate buffer.

**Fig. 2:**
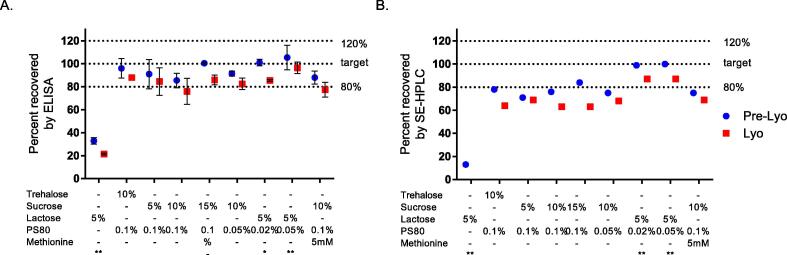
Effect of sugar and stabilizing excipients on dmLT stability. The stability of dmLT at 20 µg/mL was evaluated in varying concentrations of trehalose, sucrose, and lactose sugars with or without the addition of PS80 and methionine both before and after lyophilization by ELISA and SEC. All formulations were prepared with 50 mM NaCl, 50 mM buffer and at a pH of 7.4 except where noted with an asterisk. *Formulation contains 20 mM buffer; ** Formulation contains 53.4 mM buffer and 82 mM NaCl.

dmLT stability with alternate sugars, trehalose, and sucrose, were compared to the existing lactose sugar. dmLT was prepared at 20 µg/mL in a phosphate buffer containing varying concentrations of each of the sugars, and stability was evaluated both before and after lyophilization using ELISA and SEC methods. Trehalose- and sucrose-containing formulations showed decreased dmLT stability after lyophilization by ELISA and SEC methods (Fig. 2). These findings do not support changing from the existing lactose in formulation.

The effects of PS80 and methionine were also evaluated based on previously published work with dmLT [11]. The addition of PS80 improved dmLT stability with the least number of changes to the formulation (Fig. 2).

**Fig. 3:**
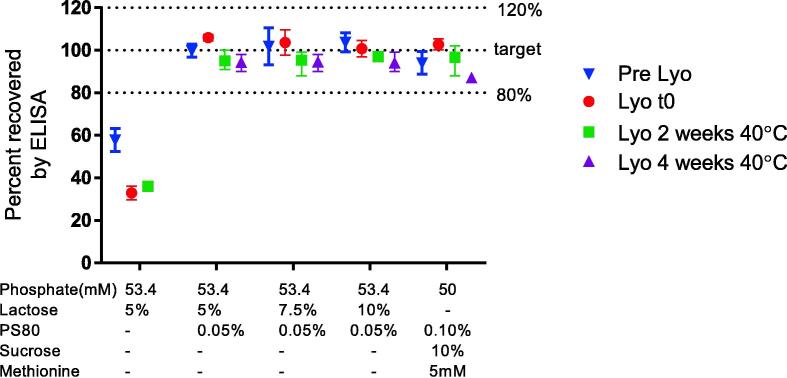
Accelerated stability of lyophilized dmLT**.** Stability of the dmLT in lyophilized formulations containing different amounts of lactose after holding at 40 °C was evaluated by ELISA. All formulations were prepared with 50 mM NaCl at a pH of 7.4.

Based on result for formulations containing phosphate buffer, lactose, and PS80, we evaluated accelerated 1-month stability of lyophilized formulations at 20 µg/mL incubated at 40 °C As seen in Fig. 3, increasing the lactose concentration beyond 5 % does not further improve the stability of dmLT. All formulations containing lactose and PS80 were able to maintain dmLT stability for 4 weeks at 40 °C.

**Fig. 4:**
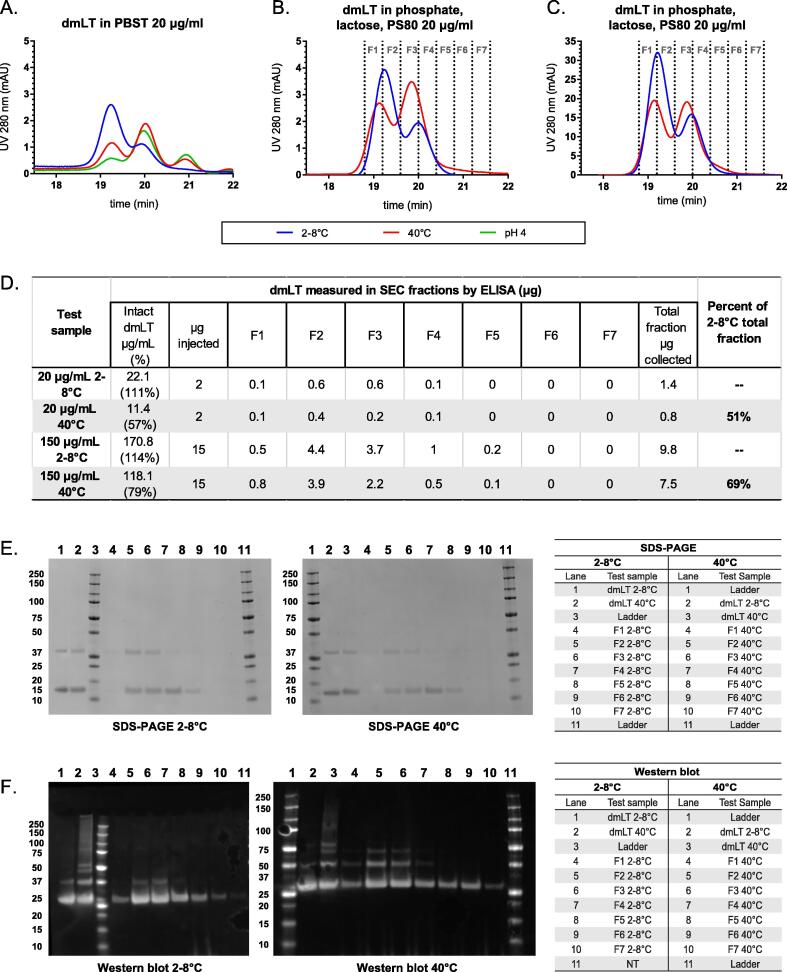
Sensitivity of *in vitro* methods to changes in dmLT stability. Chromatograms of dmLT samples subjected to heat stress (3 days at 40 °C) or low pH (4 ± 0.2) measured by UV absorbance SEC are shown in A) 20 µg/mL dmLT PBST; B) dmLT in the lead formulation (phosphate buffer with lactose and PS80) at 20 µg/mL; and C) dmLT in the lead formulation at 150 µg/mL. D) ELISA recovery of dmLT fractions 1–6 collected from SEC for 20 µg/mL dmLT samples and 150 µg/mL dmLT samples. E) SDS-PAGE gels of 150 µg/mL dmLT fractions; subunit A at 28 kDa, subunit B at 11.5 kDa. F) WB of 150 µg/mL dmLT fractions detected with an antibody against the A-subunit.

To evaluate the effect of pH (low pH ≤ 4.2) and heat stress (40 °C) on dmLT at 20 µg/mL in PBST, samples were tested after holding for 3 days prior to testing by SEC. Samples demonstrated significant loss of intact dmLT as seen in SEC (Fig. 4A). The effect of heat stress (40 °C for 3 days) was further evaluated by formulating dmLT at 20 µg/mL and 150 µg/mL in phosphate-buffered formulations containing lactose and PS80 and testing by SEC. Improved stability of intact dmLT was observed after heating for 3 days at 40 °C with the addition of PS80 (Fig. 4B and 4C). To better evaluate how dmLT was degrading due to heat stress, SEC peak fragments were collected for testing by ELISA, WB, and SDS-PAGE testing (Fig. 4D, 4E, and 4F). Loss of intact dmLT was observed in all methods after exposure to heat stress. The expected size of intact dmLT is approximately 85.5 kDa (one A-subunit 28 kDa and five B-subunits 11.5 kDa). Recovery of intact dmLT by the ELISA demonstrated approximately a 50 % difference in samples held at 2–8 °C compared to samples held at 40 °C (Fig. 4D). Slightly higher recovery of intact dmLT was observed in dmLT formulated at 150 µg/mL (69 %) compared to 20 µg/mL (51 %), which is likely due to increased dmLT stability at a higher protein concentration.

**Fig. 5:**
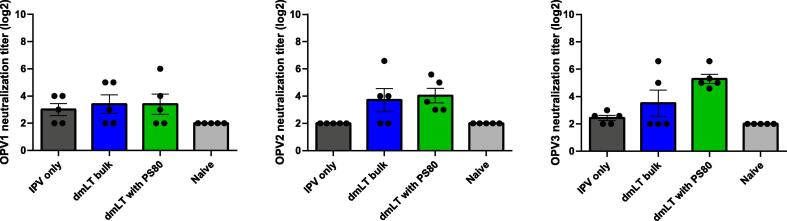
Adjuvanticity of dmLT formulation. BALB/c mice were immunized 3 times at a 3-week interval and sera were collected and tested for neutralizing antibody responses against oral poliovirus types 1, 2, 3.

The lead dmLT formulation at 20 µg/mL containing lactose and PS80 adjuvanticity was compared to existing dmLT bulk to ensure re-formulation had no impact on dmLT activity. Mice were dosed with IPV with and without either dmLT with PS80 or the bulk dmLT and neutralizing antibody responses were compared (Fig. 5). No significant differences were observed between the dmLT formulated with PS80 and the bulk dmLT. Additional animal work was conducted with dmLT formulations under stressed conditions by Tulane University. Results are being published by Dr. Norton in a separate publication.

## 4 Discussion

In this study we evaluated different formulations of dmLT to improve stability at 20 µg/mL, which limits the amount of dilution required to achieve a relevant clinical dose and limits waste. Identifying stabilizing formulations for dmLT as well as *in vitro* characterization methods are key to the further development of this adjuvant [14]. Existing dmLT stocks supply the adjuvant at 1 mg/mL while clinical studies require concentrations of 1 to 5 µg/mL. This work focused on identifying a stable formulation that would improve usability and limit waste of dmLT. In addition, this work sought to further develop *in vitro* methods for dmLT characterization.

Formulations that limited changes to the existing dmLT formulation were given preference to allow for bridging to existing toxicology and human clinical data. No significant differences were observed by changing the buffer or sugar present in the formulation. Heat-labile toxin and dmLT have been shown to bind to sugars containing a terminal galactose such as lactose [15], [16]. It is unclear how lactose binding affects dmLT activity. However, all clinical studies with dmLT have contained lactose. In this study, lactose-containing formulations maintained dmLT stability at a concentration of 20 µg/mL. In addition, lactose has been shown to reduce reactogenicity with no effect on immunogenicity in mice [19]. Further studies are needed to better understand the effect of lactose on dmLT activity. The most significant difference was observed by the addition of PS80 to the formulation, which maintained dmLT stability for 1 month at 40 °C. PS80 is a nonionic surfactant that prevents surface adsorption and protein aggregation [17]. The PS80 concentrations tested were based on either previously published studies or on the concentrations found in existing licensed vaccine products [11], [18]. Existing bulk lyophilized dmLT formulations at 1 mg/mL are stored frozen at −20 °C and have demonstrated stability for 10-years. Based on the stability data obtained in these studies the authors would expect this novel bulk dmLT formulation at 20 µg/mL to achieve a similar if not improved shelf-life potentially at a refrigerated temperature eliminating the need for frozen storage. All the work presented here focused on studies of the dmLT adjuvant stock stability. Future work is needed to better understand the effect of different antigens and matrices on dmLT stability.

## 5 Author’s contributions

MRE, AB and JAW designed and conducted the experiments. MRE, AB, JAW and EBN drafted the manuscript.

## Declaration of Competing Interest

The authors declare that they have no known competing financial interests or personal relationships that could have appeared to influence the work reported in this paper.

## Data Availability

Data will be made available on request.

## References

[1] Leach S., Clements J.D., Kaim J., Lundgren A. (2012). The adjuvant double mutant *Escherichia coli* heat labile toxin enhances IL-17A production in human T cells specific for bacterial vaccine antigens. PLoS One.

[2] Norton E.B., Lawson L.B., Freytag L.C., Clements J.D. (2011 Apr). Characterization of a mutant Escherichia coli heat-labile toxin, LT(R192G/L211A), as a safe and effective oral adjuvant. Clin Vaccine Immunol.

[3] John D Clements, Elizabeth B Norton. The mucosal vaccine adjuvant LT(R192G/L211A) or dmLT. mSphere. 2018 Jul 25;3(4):e00215-18. Doi: 10.1128/mSphere.00215-18. 10.1128/mSphere.00215-18 PMC6060342 30045966

[4] Lundgren A., Bourgeois L., Carlin N., Clements J., Gustafsson B., Hartford M. (2014 Dec 12). Safety and immunogenicity of an improved oral inactivated multivalent enterotoxigenic *Escherichia coli* (ETEC) vaccine administered alone and together with dmLT adjuvant in a double-blind, randomized, placebo-controlled Phase I study. Vaccine.

[5] Marjahan Akhtar, Nuder Nower Nizam, Salima Raiyan Basher, Lazina Hossain, Sarmin Akter, Taufiqur Rahman Bhuiyan, et al. dmLT adjuvant enhances cytokine responses to T cell stimuli, whole cell vaccine antigens and lipopolysaccharide in both adults and infants. Frontiers in Immunology. 2021 May 14;12:654872. Doi: 10.3389/fimmu.2021.654872. 10.3389/fimmu.2021.654872 PMC8160295 34054818

[6] Bernstein D.I., Pasetti M.F., Brady R., Buskirk A.D., Wahid R., Dickey M. (2019 Jan 21). A Phase 1 dose escalating study of double mutant heat-labile toxin LTR192G/L211A (dmLT) from Enterotoxigenic *Escherichia coli* (ETEC) by sublingual or oral immunization. Vaccine.

[7] Lee T., Gutiérrez R.L., Maciel M., Poole S., Kayla Testa J., Stefanie Trop (2021 Sep 15). Safety and immunogenicity of intramuscularly administered CS6 subunit vaccine with a modified heat-labile enterotoxin from enterotoxigenic *Escherichia coli*. Vaccine.

[8] Crothers J.W., Colgate E.R., Cowan K.J., Dickson D.M., Walsh MaryClaire, Carmolli M. (2022 Apr 26). Intradermal fractional-dose inactivated polio vaccine (fIPV) adjuvanted with double mutant Enterotoxigenic *Escherichia coli* heat labile toxin (dmLT) is well-tolerated and augments a systemic immune response to all three poliovirus serotypes in a randomized placebo-controlled trial. Vaccine.

[9] Qi Y., Fox C.B. (2021 May). Development of thermostable vaccine adjuvants. Expert Rev Vaccines.

[10] Jessica A White, Candace Haghighi, Johanna Brunner, Marcus Estrada, Manjari Lal, Dexiang Chen. Preformulation studies with the *Escherichia coli* double mutant heat-labile toxin adjuvant for use in an oral vaccine. Journal of Immunological Methods. 2017 Dec;451:83-89. Doi: 10.1016/j.jim.2017.09.003. 10.1016/j.jim.2017.09.003 PMC5703769 28939395

[11] Toprani V.M., Sahni N., Hickey J.M., Robertson G.A., Russell Middaugh C., Joshi S.B. (2017 Oct 4). Development of a candidate stabilizing formulation for bulk storage of a double mutant heat labile toxin (dmLT) protein based adjuvant. Vaccine.

[12] Norton E.B., Branco L.M., Clements J.D. (2015). Evaluating the A-subunit of the heat-labile toxin (LT) as an immunogen and a protective antigen against enterotoxigenic *Escherichia coli* (ETEC). PLoS One.

[13] Mudrak B., Kuehn M.J. (2010 Jun). Heat-labile enterotoxin: beyond G_M1_ binding. Toxins.

[14] Shi S., Zhu H., Xia X., Liang Z., Ma X., Sun B. (2019 May 27). Vaccine adjuvants: understanding the structure and mechanism of adjuvanticity. Vaccine.

[15] Shida K., Takamizawa K., Nagaoka M., Tsuji T., Osawa T. (1994). Escherichia coli heat-labile enterotoxin binds to glycosylated proteins with lactose by amino carbonyl reaction. Microbiol Immunity.

[16] Toprani V.M., Hickey J.M., Sahni N., Toth IV R.T., Robertson G.A., Russell Middaugh C. (2017 Aug). Structural characterization and physicochemical stability profile of a double mutant heat labile toxin protein based adjuvant. J Pharm Sci.

[17] Kerwin B.A. (2008 Aug). Polysorbates 20 and 80 used in the formulation of protein biotherapeutics: structure and degradation pathways. J Phamaceut Sci.

[18] Michael T Jones, Hanns-Christian Mahler, Sandeep Yadav, Dilbir Bindra, Vincent Corvari, R Matthew Fesinmeyer, et al. Considerations for the use of polysorbates in biopharmaceuticals. Pharmaceutical Research. 2018 May 24;35(8):148. Doi: 10.1007/s11095-018-2430-5. 10.1007/s11095-018-2430-5 29797101

[19] Maciel M Jr, Bauer D, Baudier RL, Bitoun J, Clements JD, Poole ST, Smith MA, Kaminski RW, Savarino SJ, Norton EB. Intradermal or Sublingual Delivery and Heat-Labile Enterotoxin Proteins Shape Immunologic Responses to a CFA/I Fimbria-Derived Subunit Antigen Vaccine against Enterotoxigenic Escherichia coli. Infect Immun. 2019 Oct 18;87(11). Doi: 10.1128/IAI.00460-19. 10.1128/IAI.00460-19 PMC6803349 31427449

